# Multi-environment gene interactions linked to the interplay between polysubstance dependence and suicidality

**DOI:** 10.1038/s41398-020-01153-1

**Published:** 2021-01-11

**Authors:** Renato Polimanti, Daniel F. Levey, Gita A. Pathak, Frank R. Wendt, Yaira Z. Nunez, Robert J. Ursano, Ronald C. Kessler, Henry R. Kranzler, Murray B. Stein, Joel Gelernter

**Affiliations:** 1 Department of Psychiatry, Yale School of Medicine, Yale University, West Haven, CT USA; 2 Veteran Affairs CT Healthcare System, West Haven, CT USA; 3 Center for the Study of Traumatic Stress, Department of Psychiatry, Uniformed Services University of the Health Sciences, Bethesda, MD USA; 4 Department of Health Care Policy, Harvard Medical School, Boston, MA USA; 5 University of Pennsylvania Perelman School of Medicine, Philadelphia, PA USA; 6 Crescenz Veterans Affairs Medical Center, Philadelphia, PA USA; 7 Department of Psychiatry, School of Medicine, University of California, San Diego, La Jolla, CA USA; 8 Psychiatry Service, Veterans Affairs San Diego Healthcare System, San Diego, CA USA; 9 Departments of Genetics and Neuroscience, Yale University School of Medicine, New Haven, CT 06510 USA

**Keywords:** Genetics, Addiction

## Abstract

Substance dependence diagnoses (SDs) are important risk factors for suicidality. We investigated the associations of multiple SDs with different suicidality outcomes, testing how genetic background moderates these associations. The Yale-Penn cohort (*N* = 15,557) was recruited to investigate the genetics of SDs. The Army STARRS (Study to Assess Risk and Resilience in Servicemembers) cohort (*N* = 11,236) was recruited to evaluate mental health risk and resilience among Army personnel. We applied multivariate logistic regression to investigate the associations of SDs with suicidality and, in the Yale-Penn cohort, we used the structured linear mixed model (StructLMM) to study multivariate gene–environment interactions. In Yale-Penn, lifetime polysubstance dependence was strongly associated with lifetime suicidality: having five SDs showed an association with suicidality, from odds ratio (OR) = 6.77 (95% confidence interval, CI = 5.74–7.99) for suicidal ideation (SI) to OR = 3.61 (95% CI = 2.7–4.86) for suicide attempt (SA). In Army STARRS, having multiple substance use disorders for alcohol and/or drugs was associated with increased suicidality ranging from OR = 2.88 (95% CI = 2.6–3.19) for SI to OR = 3.92 (95% CI = 3.19–4.81) for SA. In Yale-Penn, we identified multivariate gene–environment interactions (Bayes factors, BF > 0) of SI with respect to a gene cluster on chromosome 16 (*LCAT*, *p* = 1.82 × 10^–7^; *TSNAXIP1*, *p* = 2.13 × 10^−7^; *CENPT*, *p* = 2.32 × 10^−7^; *PARD6A*, *p* = 5.57 × 10^−7^) for opioid dependence (BF = 12.2), cocaine dependence (BF = 12.1), nicotine dependence (BF = 9.2), and polysubstance dependence (BF = 2.1). Comorbidity of multiple SDs is a significant associated with suicidality and heritability of suicidality is partially moderated by multivariate gene interactions.

## Introduction

Individuals with substance dependence diagnoses (SDs) are a population with high suicide risk. Compared to the general population, people with SDs are 10 to 14 times as likely to die by suicide and poly-drug abusers have 17-fold increased risk of suicide rates^1^. In a large study conducted among individuals receiving Veterans Health Administration (VHA) care (fiscal years 2005–2006, *N* = 4,863,086), current diagnoses of alcohol, cocaine, cannabis, opioid, amphetamine, and sedative use disorders were all associated significantly with increased risk of suicide mortality^2^. Among people who report fair or poor health on the National Survey of Drug Use and Health (2006–2014; *N* = 502,467), those who had DSM-IV (Diagnostic and Statistical Manual of Mental Disorders, 4th Edition) alcohol use disorders, painkiller use disorders, both alcohol and marijuana use disorders, and both alcohol and cocaine use disorders were 2.72 times (95% confidence interval, CI = 1.81–4.09), 2.25 times (95% CI = 1.04–4.90), 2.38 times (95% CI = 1.25–4.54), and 3.15 times (95% CI = 1.16–8.60) as likely as people without SD to attempt suicide, respectively^3^. Although these data support high comorbidity between suicidality and SDs, very limited information is available regarding the underlying molecular mechanisms.

Recently, genome-wide association studies (GWAS) of suicidality have been conducted in large cohorts, identifying several risk loci and strong genetic overlap with depression^4–8^. However, to our knowledge, no gene-by-environment genome-wide interaction studies (GEWIS) have yet been conducted to evaluate the genetic interplay between suicidality and SDs. In the context of gene-by-environment interaction studies, environmental risk factors can include exposures (physical, chemical, biological), behavioral patterns, or life events^9^. We previously showed that this approach is useful to identify the complex interactive mechanisms linking genetic background with the interplay between SDs and psychiatric and behavioral phenotypes^10–13^. To explore the phenotypic association of different SDs with the suicidality spectrum, we investigated multiple SDs (testing for each SD the individual effect independent from the other SDs; and the cumulative effect of having received multiple SD diagnoses) with respect to suicidal ideation (SI), suicide planning (SP), and suicide attempt (SA). Subsequently, we conducted a discovery GEWIS to verify whether SDs interact with the individual genetic variability in the context of SI. Finally, based on the high genetic overlap between suicidality and depression observed across multiple large-scale GWAS^4,7,8^, we verified whether depression genetic risk interacts with polysubstance dependence in the context of suicidality spectrum. Supplemental Fig. 1 provides a graphical overview of the analyses conducted.

## Materials and methods

### Study populations

Yale-Penn participants were recruited for studies of the genetics of drug or alcohol dependence in five eastern U.S. centers as described elsewhere^14–16^. Subjects gave written informed consent as approved by the institutional review board at each site. Subjects were evaluated with the semi-structured assessment for drug dependence and alcoholism (SSADDA) to derive diagnostic and statistical manual of mental disorders, fourth edition (DSM-IV) lifetime SD diagnoses, and other major psychiatric traits. In the present study, we used information regarding DSM-IV SD diagnoses and criterion counts related to alcohol dependence (AD), cannabis dependence (CaD), cocaine dependence (CoD), nicotine dependence (ND), and opioid dependence (OD). Detailed information regarding these phenotypic definitions is provided in our previous studies^14–16^. Data regarding suicidality outcomes were derived from SSADDA items: SI “*Have you ever thought about killing yourself?* [Yes/No]*”*; persistent SI “*Did those thoughts persist for at least 7 days in a row?* [Yes/No]”; SP “*Did you have a plan?* [Yes/No]”; and SA “*Have you ever tried to kill yourself?* [Yes/No]”. Individuals not reporting SI were not asked about persistent SI and SP. Accordingly, individuals who did not endorse SI were also coded as not having persistent SI and SP. However, regardless of reporting SI, persistent SI, and SP, all participants were asked if they had ever attempted suicide. This is because SA can be an impulsive behavior with no previous ideation and planning. Suicidality and polysubstance dependence phenotypic information was available for 15,557 Yale-Penn participants. Full genome-wide data were available for a subset (~10,000) of these individuals via genotyping done with the Illumina HumanOmni1-Quad microarray, the Illumina HumanCoreExome array, or the Illumina Multi-Ethnic Global Array. Principal component (PC) analysis was conducted based on each genotyping array and for each ancestry group (African and European ancestries) separately. Detailed information about the quality control pipeline is available in our previous studies^14–16^. Briefly, Individuals and SNPs with genotype call rates <98%, and SNPs with minor allele frequency <1% and Hardy–Weinberg equilibrium *P* < 1 × 10^−6^ were removed from downstream analyses. After the pre-imputation quality control, genotype data were imputed using Minimac3^17^ implemented in the Michigan Imputation Server (available at https://imputationserver.sph.umich.edu/) with the 1000 Genomes Phase 3 reference panel^18^. Dosage data were transformed into best-estimate genotypes using PLINK2^19^, considering variants with info score ≥80% and minor allele frequency ≥1%. In the Yale-Penn participants of African and European descent, we investigated 4,915,647 and 4,202,333 variants, respectively. The present study only considered information regarding unrelated subjects. As previously described^20^, individuals with an identity-by-descent proportion >0.125 were defined as belonging to the same family group. Within each family group determined from genetic data, a subject was selected prioritizing retention of participants who reported the most extreme suicidality among those genetically related.

The Army STARRS (Study to Assess Risk and Resilience in Servicemembers) participants included individuals recruited from two different groups: The New Soldier Study and The Pre-Post Deployment Study. All subjects gave written informed consent to participate. These procedures were approved by the Human Subjects Committees of all collaborating institutions. Detailed information about the design and conduct of the Army STARRS is available in a previous report^21^. Every individual was diagnosed using a self-administered questionnaire, which included the adapted versions of the Composite International Diagnostic Interview Screening Scales (CIDI-SC). As previously described^22^, the CIDI‐SC assessment was used to determine lifetime prevalence of 12 common lifetime DSM-IV mental disorders, including substance use disorder (substance dependence and abuse) for alcohol and/or drugs combined (i.e., SUD_combined_). Suicidality was assessed using a modified version of the Columbia–Suicide Severity Rating Scale, which assesses the lifetime occurrence of SI (“*Did you ever in your life have thoughts of killing yourself*? [Yes/No]” OR “*Did you ever wish you were dead or would go to sleep and never wake up?* [Yes/No]”), SP (“*Did you ever have any intention to act [on these thoughts/on that wish*]? [Yes/No]” AND, if so, (“*Did you ever think about how you might kill yourself [e.g., taking pills, shooting yourself] OR work out a plan of how to kill yourself?* [Yes/No]”)), and SA (“*Did you ever make a suicide attempt [i.e., purposefully hurt yourself with at least some intention to die]?* [Yes/No]”). All respondents who reported ideation (regardless of intent/plan) were asked if they had ever attempted suicide. SA was considered present if respondents endorsed ever purposefully hurting themselves with at least some intention to die. Army STARRS participants were genotyped using the Illumina OmniExpress and Exome array or the Illumina PsychChip array. Methods for quality control, imputation, ancestry assignment and PC analysis were described previously^23^. Briefly, the quality control parameters applied included SNP missingness <0.05, subject missingness <0.02; autosomal heterozygosity deviation; SNP missingness of <0.02; and deviation from Hardy–Weinberg equilibrium *P* < 1 × 10^−6^. Genotyped data were pre-phased using SHAPEIT^24^ and imputed using IMPUTE2^25^ and the 1000 Genomes Project reference panel^26^. Unrelated individuals were identified considering an identity-by-descent proportion <0.125.

Table 1 reports the characteristics of the Yale-Penn and Army STARRS participants investigated in the present study. Although Yale-Penn and Army STARRS present different assessments and characteristics, the data from these cohorts were successfully used to replicate genetic associations identified in studies of SDs and suicidality^8,11^.

**Table 1:** Characteristics of the Yale–Penn and Army STARRS participants investigated in the present study.

Yale-Penn, *n* = 15,557	
Age, mean (SD)	40 (11.8)
Sex, Women (%)	7187 (46)
Self-reported Racial/Ethnic Group, *n* (%)	
Native American/American Indian	1327 (9)
Asian	101 (1)
Pacific Islander	20 (<1)
African-American/Black, not of Hispanic origin	6027 (39)
African-American/Black, of Hispanic origin	350 (2)
Caucasian/White, not of Hispanic origin	6060 (39)
Caucasian/White, of Hispanic origin	811 (5)
Other	861 (6)
DSM-IV diagnosis, *n* (%)	
Alcohol Dependence	7481 (48)
Cannabis Dependence	3897 (25)
Cocaine Dependence	8662 (56)
Nicotine Dependence	8219 (52)
Opioid Dependence	4379 (28)
Polysubstance dependence, *n* (%)	
One DSM-IV SD diagnosis	2023 (13)
Two DSM-IV SD diagnoses	2942 (22)
Three DSM-IV SD diagnoses	3345 (22)
Four DSM-IV SD diagnoses	2419 (16)
Five DSM-IV SD diagnoses	1004 (6)
Suicidality, *n* (%)	
Ideation	6112 (39)
Persistent Ideation	1450 (9)
Planning	2491 (16)
Attempt	1965 (13)

Army STARRS, *n* = 11,235	
Age, mean (SD)	21 (5.2)
Sex, Women (%)	1163 (10)
SUD_combined_, *n* (%)	2848 (22)
Suicidality, *n* (%)	
Ideation	2299 (20)
Planning	446 (4)
Attempt	389 (3)

### Data analysis

#### Phenotypic associations

We used multivariate logistic regression models to test the association of SDs with suicidality outcomes (i.e., SI, SP, and SA). In the Yale-Penn cohort, this analysis was conducted in the full sample (*N* = 15,557), which included genotyped and non-genotyped individuals. Accordingly, the following covariates were considered: age, sex, and self-reported racial/ethnic groups. In the Army STARRS cohort, the logistic regression models were applied to a fully genotyped sample of participants of European descent (*N* = 11,235) and covariates considered were: age, sex, and the top 10 genetic PCs for population stratification adjustment. The different approaches used in Yale-Penn and Army STARRS cohorts are due to the sample characteristics and the data availability. The Yale-Penn cohort includes more than 15,000 participants but, to date, ~10,000 individuals have genome-wide data available. Approximately 80% of Yale–Penn participants report being Caucasian/White or African-American/Black not of Hispanic origin (Table 1). To avoid excluding individuals without genotype information or belonging to a racial/ethnic group not large to be analyzed separately, we decided to analyze Yale-Penn combining the full sample and correcting for self-reported racial/ethnic groups. The characteristics of the Yale–Penn participants stratified by the inclusion in the genetic analyses are reported in Supplemental Table 1.

#### Genome-wide Gene-by-SD interaction analysis of SI

In the Yale–Penn cohort, we conducted a multivariate GEWIS considering unrelated participants with complete genotype information (4,044 African-Americans and 3,407 European-Americans). The analysis was conducted using the recently-developed StructLMM, a linear mixed-model approach to identify and characterize loci that interact with one or more environments efficiently^27^. This method extends the conventional linear mixed models used to test persistent genetic effects (i.e., associations with constant genetic effect sizes across individuals in the population), permitting the investigator to model the heterogeneity in effect sizes due to gene-by-environment interactions. The multi-environment StructLMM model can be used to conduct an interaction test and an association test^27^. The interaction test is defined where persistent genetic and additive environment effects are accounted for in the null model. Conversely, the StructLMM association test analyzes the main effects while accounting for the possibility of heterogeneous genetic effects due to G × E.

We used the StructLMM approach to analyze whether DSM-IV criterion counts of AD, CaD, CoD, OD, and ND and the co-occurrence of multiple DSM-IV SD diagnoses interact at the same loci with respect to SI. We limited the genetic analysis to SI, because of the relatively low prevalence of the other suicidality outcomes in the Yale-Penn cohort (Table 1). From the StructLMM framework, we obtained evidence of: (i) loci with significant SD-related interaction effects and (ii) genetic association accounting for the possibility of heterogeneous effect sizes due to multivariate SD–gene interactions. P values were used to verify the statistical significance of the association and interaction tests in each locus. Once we identified the loci surviving multiple-testing correction, StructLMM permitted us to calculate Bayes factors (BF) to interpret evidence for environment relevance, including potential for positive and negative BFs supporting the alternate *vs*. null models, respectively. Specifically, the BFs were calculated between the full model and models with environmental variables removed to identify which SD-related traits are most relevant for the gene interactions observed. Additionally, we also estimated the fraction of genetic variance explained by multivariate SD-gene interactions. These analyses were conducted separately in each major genetically-determined ancestry group (i.e., African-Americans and European-Americans). We focused our analysis on ancestry-specific analyses only, because the trans-ancestry meta-analysis did not provide findings surviving multiple testing correction due to the fact that the limited sample size of the cohorts investigated was not powerful enough to overcome the heterogeneity due to the different genetic structure (i.e., allele frequency and linkage disequilibrium, LD) of the ancestry groups investigated. The information regarding SI was adjusted for age, sex, genotyping array, and the top 10 PCs, and the residuals obtained were entered as phenotypic outcomes into StructLMM.

To increase the discovery power of the analysis conducted in the Yale-Penn cohort, we used the single-variant results obtained from the StructLMM interaction and association tests to conduct genome-wide gene-based analyses considering interactive and main effects, respectively. We applied the Multi-marker Analysis of GenoMic Annotation (MAGMA) gene-based approach^28^ and a Bonferroni multiple testing correction. Gene-based tests are generally more powerful than single-variant association analysis^28^, because they combine single-variant signals within genic regions reducing the multiple testing correction burden. We performed a functional annotation of the variants identified using data from combined annotation dependent depletion (CADD)^29^, RegulomeDB^30^, and 15-core chromatin state information across multiple brain tissues. Using genotype-tissue expression (GTEx) V8^31^, we tested the effect of the variants identified on the tissue-specific transcriptomic profiles of the surrounding genes (±1 Mb of the gene transcription starting site), considering a false discovery rate at 5% for the genome-wide multiple testing correction. To investigate the loci identified further, we performed single-variant and gene-based phenome-wide scans leveraging the GWAS Atlas (available at https://atlas.ctglab.nl/)^32^.

#### Replication analysis

The Army STARRS cohort (11,235 participants of genetically-confirmed European descent; see *Study Populations*) was investigated to replicate the Yale–Penn finding on chromosome 16 (i.e., rs8052287). Due to the unavailability of information about multiple SDs among the participants, we tested the gene interaction of SUD_combined_ (i.e., a single composite variable combining substance use disorders for alcohol and/or drugs) with respect to SI. This analysis was conducted using the interaction test available in PLINK 1.9^19^, which compares the difference between SI regression coefficients in subjects with SUD_combined_ vs. those without SUD_combined_. Age, sex, and the top 10 PCs were entered as covariates.

#### Polygenic risk score analysis

We leveraged the Psychiatric Genomic Consortium (PGC) GWAS of major depression (MD)^33^ to generate polygenic risk scores (PRS) in Yale-Penn participants of European descent (*N* = 3,407). We focused our attention on MD because of the consistent genetic overlap of this trait with suicidality^4,7,8^. Due to the data sharing restrictions of the 23andMe personal genomics and biotechnology company (a contributor to the PGC MD cohort), GWAS data were publicly available only for a sample subset (59,851 MD cases and 113,154 controls). This analysis was restricted to the participants of genetically confirmed European descent due to known biases of cross-ancestry PRS analysis^34^. The MD PRS was calculated using PLINK 1.9^19^, considering multiple association *P*-value thresholds (PT < 5 × 10^−8^, 10^−7^, 10^−6^, 10^−5^, 10^−4^, 0.001, 0.05, 0.1, 0.3, 0.5, 1) for SNP inclusion to identify the best-fit for each target phenotype tested. The PRS were calculated after using *P*-value-informed clumping with a LD cut-off of R^2^ = 0.3 within a 500 kb window and excluding the major histocompatibility complex region of the genome because of its complex LD structure. The individual PRS generated were standardized and entered into a logistic regression model that included the main effect (PRS) and the effect of the interaction term (i.e., the product of PRS and the covariate for interaction). A false discovery rate (FDR q < 0.05) was applied to correct the PRS results for the number of thresholds and phenotypes tested.

## Results

### Phenotypic associations

**Fig. 1:**
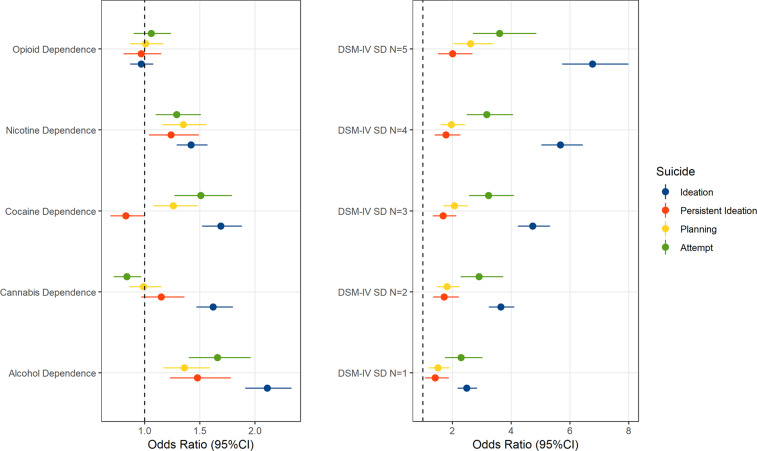
Substance dependence (SD) and suicidality in Yale-Penn participants. Association of DSM-IV SD diagnoses (left panel) or polysubstance dependence severity (i.e., number of comorbid DSM-IV SD diagnosis; right panel) with suicidal ideation, persistent ideation, planning, and attempt.

To identify effects accounting for the comorbidity among the SDs tested in the Yale-Penn cohort, AD, CaD, CoD, ND, and OD were entered as terms in the same logistic regression model (Fig. 1, left panel). We observed a consistent effect of AD and ND across all suicide traits tested, ranging from AD OR = 2.11 (95% CI = 1.91–2.33) and ND OR = 1.42 (95% CI = 1.29–1.57) for SI; to AD OR = 1.66 (95% CI = 1.4–1.96) and ND OR = 1.29 (95% CI = 1.1–1.51) for SA. Increased odds were observed with respect to CoD for SI (OR = 1.69; 95% CI = 1.52–1.88), SP (OR = 1.26; 95% CI = 1.08–1.48), and SA (OR = 1.51; 95% CI = 1.27–1.79), but not for persistent SI. CaD showed opposite effect directions for these traits; positive association with SI (OR = 1.62; 95% CI = 1.47–1.80) and negative association with SA (OR = 0.84; 95% CI = 0.72–0.97). An additional regression analysis was conducted to investigate the association of the severity of polysubstance dependence (i.e., the number of SD diagnoses) with suicidality (Fig. 1, right panel). Considering the most extreme cases (i.e., all 5 SDs), we observed the largest effects: OR = 6.77 (95% CI = 5.74–7.99) for SI; OR = 2.01 (95% CI = 1.51–2.68) for persistent SI; OR = 2.62 (95% CI = 2.04–3.39) for SP; OR = 3.61 (95% CI = 2.7–4.86) for SA. The Yale-Penn cohort includes individuals reporting different racial/ethnic groups (Table 1). We observed that self-reported racial/ethnic groups were associated with suicidality outcomes when adjusted for age, sex, and polysubstance dependence (Supplemental Tables 2 and 3). In the Army STARRS participants (*N* = 11,236), we observed similar effects of SUD_combined_: OR = 2.88 (95% CI = 2.6–3.19) for SI; OR = 3.88 (95% CI = 2.79–4.10) for SP; OR = 3.92 (95% CI = 3.19–4.81) for SA.

### Multivariate SD–gene interaction analysis in participants of european descent

**Fig. 2:**
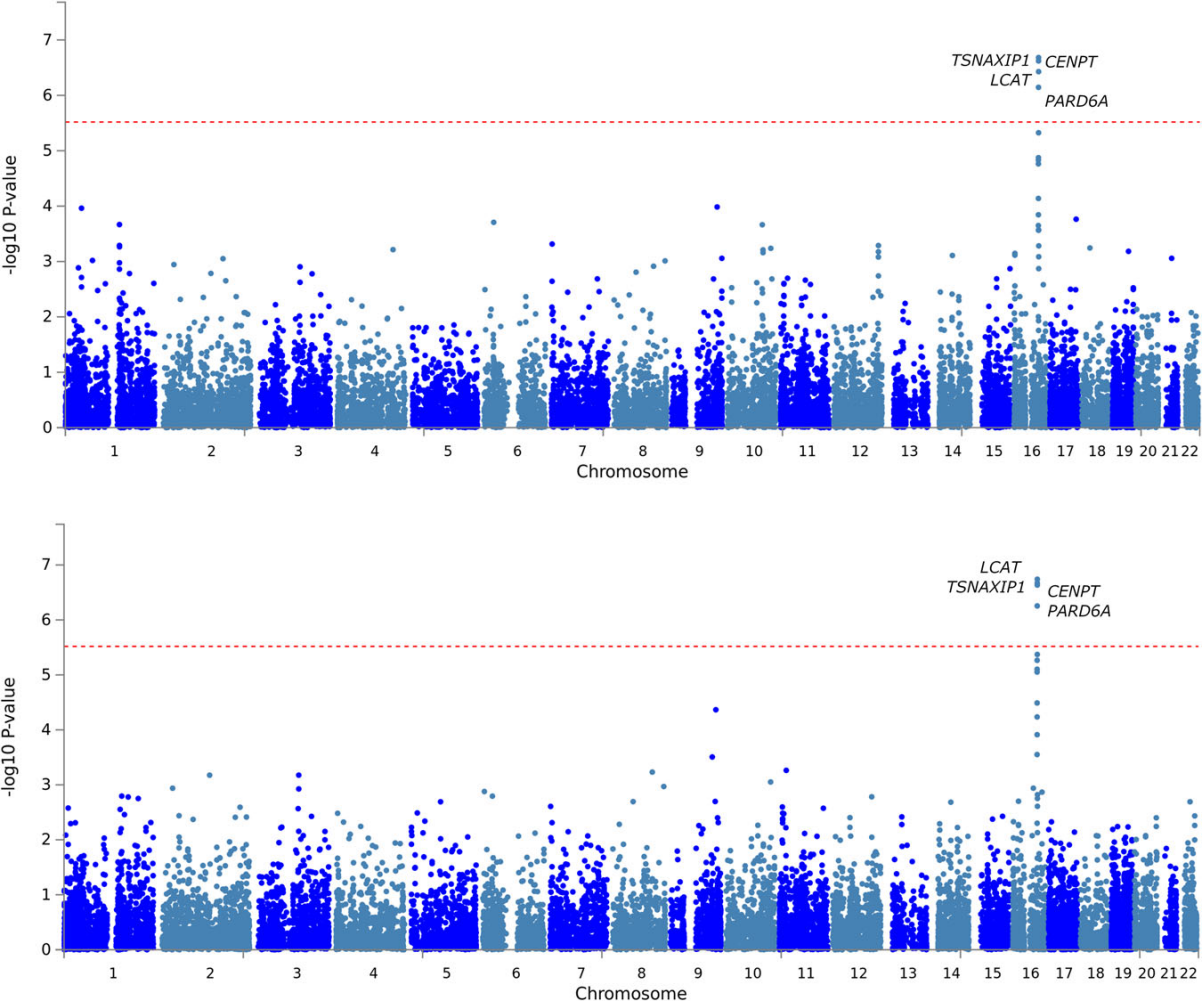
Gene-based Manhattan plots generated from the multivariate GEWIS of suicide ideation. Bottom panel: interactive effects where persistent genetic and additive environment effects are accounted for in the null model; Top panel: association effects accounting for the heterogeneous effect size due to the interactive effects). Red dashed line represents the significance threshold accounting for the gene-based Bonferroni multiple testing correction.

**Fig. 3:**
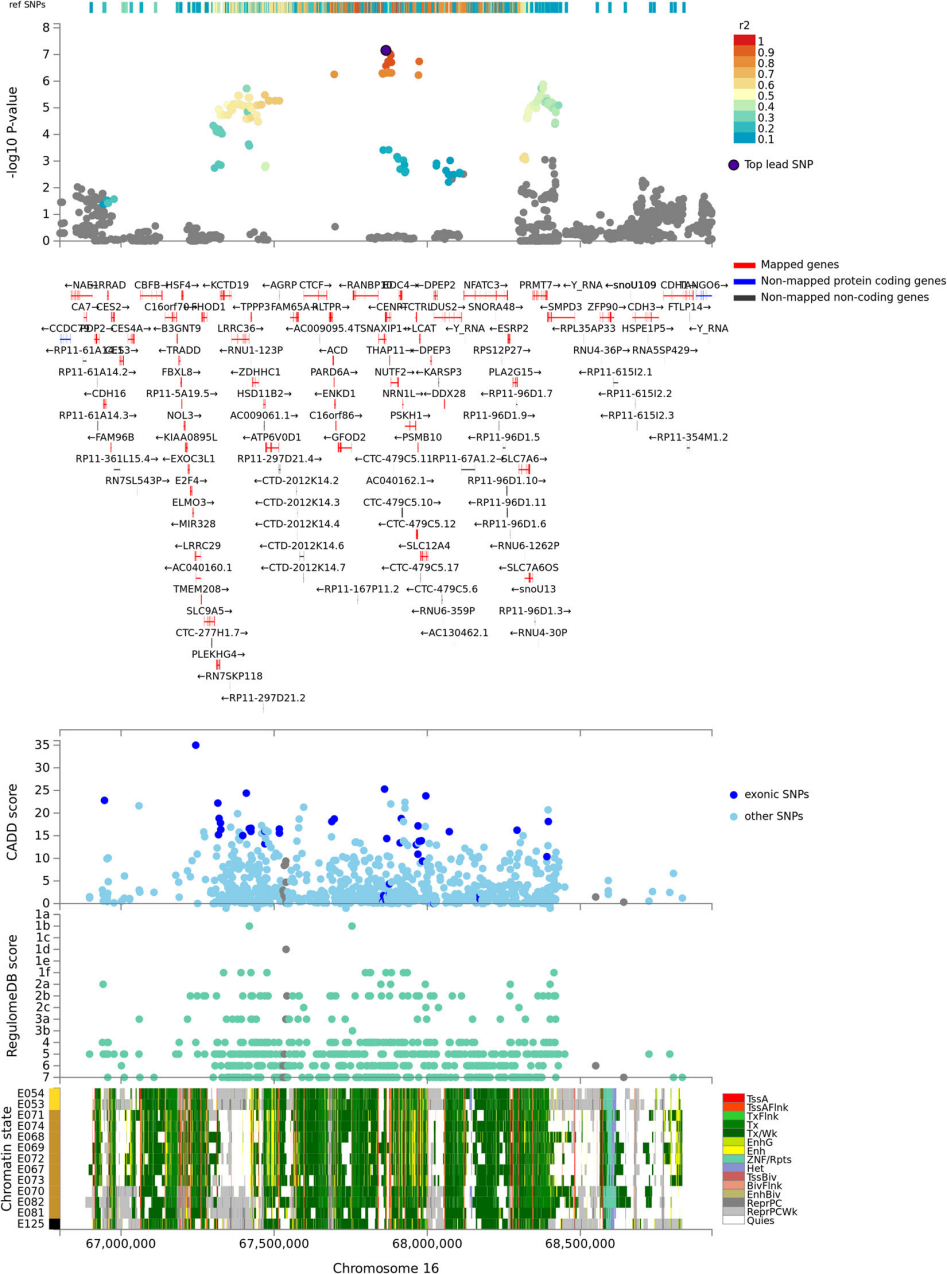
Regional Manhattan plot of the lead variant rs8052287. This was identified in the gene-based multivariate GEWIS of suicide ideation (Yale-Penn participants of European descent) Functional annotation derived from CADD (Combined Annotation Dependent Depletion) and RegulomeDB scores and 15-core chromatin state information across 13 brain tissues is included below. CADD scores > 20 corresponds to top-1% of pathogenicity across the human genome. RegulomeDB Score = 1 (f to a) corresponds to variants located within a transcription factor binding that shows eQTL activity.

Among Yale-Penn participants of European descent, several genes on chromosome 16 survived multiple testing correction for both association and interactive effects (Fig. 2): *LCAT* (p_association_ = 3.73 × 10^−7^; p_interaction_ = 1.82 × 10^−7^); *TSNAXIP1* (p_association_ = 2.08 × 10^−7^; p_interaction_ = 2.13 × 10^−7^), *CENPT* (p_association_ = 2.39 × 10^−7^; p_interaction_ = 2.32 × 10^−7^), and *PARD6A* (p_association_ = 7.17 × 10^−7^; p_interaction_ = 5.57 × 10^−7^). The association of this gene cluster is driven by the effect of a single variant, rs8052287 (p_association_ = 2.15 × 10^−7^; p_interaction_ = 7 × 10^−8^; Fig. 3). Within this locus, 98% of the variance is explained by multivariate SD-gene interactions. We calculated the Bayes factors (BF) between the full model and models including the individual environmental exposures removed to explore which environmental variables are most relevant for the gene-environment signals of rs8052287. We observed putative gene-environment effects in rs8052287 (BF > 0) for OD criterion counts (BF = 12.2), CoD criterion counts (BF = 12.1), ND criterion counts (BF = 9.2), and co-occurrence of multiple SD diagnoses (BF = 2.1). We found that rs8052287 was associated with the transcriptomic regulation of 26 genes in 30 different tissues (Supplemental Table 4). Considering brain tissues, we observed that rs8052287 is associated with *RANBP10* in the cerebellum (*p* = 6.2 × 10^−18^), cortex (*p* = 4.3 × 10^−12^), frontal cortex (BA9; *p* = 4.9 × 10^−9^), cerebellar hemisphere (*p* = 8.9 × 10^−9^), caudate (*p* = 4.8 × 10^−7^), and nucleus accumbens (*p* = 2.5 × 10^−5^). Rs8052287 is also a splicing quantitative trait locus for *CARMIL2* in the frontal cortex (BA9; *p* = 1.2 × 10^−6^). Considering data available from the GWAS atlas, we observed 20 significant associations that survived phenome-wide multiple testing correction (Supplemental Table 5), including anthropometric traits (e.g., height *p* = 8.30 × 10^−22^), male hair loss pattern (*p* = 1.11 × 10^−10^), hypothyroidism (*p* = 5.97 × 10^−7^), and several hematological parameters (e.g., mean corpuscular hemoglobin *p* = 4.74 × 10^−7^). The same associations were also observed in the gene-based phenome-wide scans conducted for *LCAT*, *TSNAXIP1*, *CENPT*, and *PARD6A* (Supplemental Table 6). We also observed that rs8052287 regulates the expression of 9 genes in thyroid tissue: *LRRC36* (*p* = 1.6 × 10^−16^), *ZDHHC1* (*p* = 3.9 × 10^−15^), *RANBP10* (*p* = 3.7 × 10^−14^), *HSD11B2* (*p* = 5.7 × 10^−9^), *C16orf86* (*p* = 3.2 × 10^−8^), *KCTD19* (*p* = 8.3 × 10^−8^), *DUS2* (*p* = 6.1 × 10^−6^), *ACD* (*p* = 9.7 × 10^−5^), *FHOD1* (*p* = 2.8 × 10^−4^). In addition to its effect on transcriptomic regulation, rs8052287 is in high LD (r^2^ = 0.81) with rs62620177, a coding variant with a CADD score of 25.3, which indicates pathogenicity in the top 1% of all SNPs in the human genome^29^.

Due to the limited phenotypic information available in the Army STARRS cohort, which consisted only of a single composite SD variable (SUD_combined_), we could not apply the StructLMM approach. Testing SUD_combined_ as a single factor, no interaction of rs8052287 was observed with respect to SI outcome (*p* > 0.05).

### Multivariate SD–gene interaction analysis in participants of african descent

In the Yale-Penn participants of African descent, we observed the *HGF* gene survived Bonferroni multiple testing correction for the StructLMM interaction test (*p* = 1.08 × 10^−6^) and approached significance in the association test (*p* = 3.39 × 10^−6^). Within the *HGF* gene region, we did not observe a driving single-variant association/interaction (all *p* > 10^−5^). Although the phenome-wide scan did not show any association surviving multiple testing correction (*p* = 1.05 × 10^−5^), the strongest *HGF* association was with one of the traumatic events assessed in the UK Biobank^35^, Data-Field 20526: “*Been in serious accident believed to be life-threatening*” (*p* = 1.21 × 10^−5^).

### Major depression polygenic risk score analysis

To follow up the consistent genetic overlap identified previously^4–8^, we focused our attention on MD PRS from a large-scale MD GWAS conducted by PGC investigators^33^. In the Yale-Penn cohort, the MD PRS was positively associated with suicidality (Supplemental Table 7): persistent SI (Yale-Penn OR = 1.26, 95% CI = 1.09–1.46, FDR q = 0.011), SP (Yale-Penn OR = 1.28, 95% CI = 1.13–1.47, FDR q = 0.005), and SA (Yale-Penn OR = 1.26, 95% CI = 1.09–1.45, FDR q = 0.011). However, there was no interaction between MD PRS and SD-related traits with respect to suicidality phenotypes (Supplemental Table 8).

## Discussion

### Phenotypic associations

Leveraging the deep phenotypic assessment available in the Yale-Penn cohort, we investigated the effects of different SDs on suicidality. AD and ND were positively associated with all suicidality outcomes tested in line with previous studies and meta-analyses^36,37^. In contrast, the other SDs investigated appear not to have the same effect across suicidality spectrum but rather specifically affect certain outcomes. The most intriguing results are related to CaD, which was associated positively with SI, negatively with SA, and a null effect was observed with respect to persistent SI and SP. A meta-analysis of studies on cannabis use and suicidality showed a positive association of cannabis use with SI, SA, and death by suicide^38^. Our analysis was based on a comprehensive assessment of multiple SDs including CaD, and, for that specific trait, the results are controlled for the effect of other SDs on suicidality. These methodological differences could explain the inconsistency between our findings and those of the previous meta-analysis. Further, the previous meta-analysis focused on varying degrees of cannabis use (any, chronic, and heavy cannabis use), while our analysis investigated CaD. The genetic basis of substance use, substance abuse, and SDs appears to be partially distinct^39–42^ and these differences may affect their associations with suicidality. With respect to this issue, an interesting approach for future investigations would be to investigate the association of SD and SUD diagnostic criteria with suicidality.

We also observed that individuals with increasing number of SD diagnoses had a larger effect on suicidality than that observed with respect to single SDs. The strongest associations were observed for SI (individuals with all five SD diagnoses showed a 6.77-fold increase in SI odds) and SA (individuals with all five SD diagnoses showed a 3.61-fold increase in SA odds) and relatively weaker associations were present for persistent SI (individuals with all five SD diagnoses showed a 2.01-fold increase in persistent-SI odds) and SP (individuals with all five SD diagnoses showed a 2.62-fold increase in SP odds). Our analysis in the Army STARRS participants also showed that SUD_combined_ is positively associated with suicidality (SI OR = 2.88; SP OR = 3.38; SA OR = 3.92).

#### Genetic findings

We conducted a multivariate gene-based GEWIS of SI, testing the interactive effect of SD-related traits. In participants of European descent, we identified multiple genes within the same region of chromosome 16 that showed both significant SD-related interaction effects and a significant association with SI accounting for the possibility of heterogeneous effect sizes due to multivariate SD–gene interactions. Investigating the index variant, rs8052287, we characterized the signal and observed that the multivariate interactions were related to DSM-IV OD, CoD, and ND criterion counts and the severity of polysubstance dependence (i.e., the number of SD diagnoses). These gene-environment interactions account for 98% of the genetic variance within this locus. This is in line with the expected statistical power of the StructLMM method, which has greater power to detect loci with a high fraction of the genetic variance explained by gene-environment interactions^27^. We could not apply the StructLMM method to the Army STARRS data because of the lack of high-dimensional data on polysubstance dependence. Applying a standard gene-environment test, we did not observe an interaction between rs8052287 and SUD_combined_ with respect to SI. This may be due to the reduction in the statistical power of standard gene-environment tests compared to the StructLMM approach^27^.

To validate our findings, we investigated the phenome-wide spectrum associated with this locus and the regulatory effect of rs8052287 (i.e., the index variant) on the tissue-specific transcriptomic profile of the genes located in this region. Gene-based and single-variant phenome-wide scans showed a similar pattern of associations related to physical health and characteristics. These included hypothyroidism, anthropometric traits, male hair loss, and hematological parameters. These phenotypic associations can be linked to altered thyroid function^43^. Tissue-specific transcriptomic analysis confirmed that rs8052287 actively regulates multiple genes in thyroid tissues. Due to the high gene density in the locus identified (rs8052287 regulates the transcriptomic profile of 26 genes), it is hard to pinpoint the gene(s) responsible for the interaction with polysubstance dependence. However, the evidence leading to altered thyroid function supports an intriguing hypothesis. Previous studies highlighted the potential role of thyroid dysfunction in suicide risk, especially among psychiatric patients^44^. This is in line with the known effect of altered thyroid function on mental health^43^. In a recent genome-wide analysis, we observed that hypothyroidism is genetically correlated with several behavioral traits including fatigue, anxiety, depression, loneliness, and mood swings^45^. Based on these findings, we hypothesize that the region identified may affect suicide risk via its role in regulating thyroid function. Additionally, the three substances (cocaine, nicotine, and opioids) that showed an interactive effect with rs8052287 on SI have been investigated previously with respect to thyroid homeostasis. Cigarette smoking appears to affect thyroid function with a dose-related effect linking cotinine levels to thyroid function and thyroid autoimmunity^46^. Cocaine abuse has been linked to the disruptions in the hypothalamic-pituitary-thyroid axis^47^ and cocaine use has been suggested as a possible trigger for thyroid storm^48^. There is a growing literature on the effect of opioids on the endocrine system and opioid-induced endocrinopathies^49^. Acute administration of various opioids has been shown to alter thyroid-stimulating hormone and thyrotropin-releasing hormone consistenly^49^. However, conflicting results were obtained by studies investigating thyroid function in long-term opioid users and controls^49^. Although the exact pathway by which the combination of CoD, ND, and OD interacts with rs8052287 in the context of SI is unclear, our “thyroid” hypothesis provides a potential pathogenetic mechanism linking polysubstance dependence, genetic liability to hypothyroidism, and SI. Further studies are needed to test this hypothesis, which has some obvious potential therapeutic implications.

In the Yale-Penn participants of African descent, we identified the *HGF* gene as an interactive locus with polysubstance dependence in the context of SI. Although we could only conduct limited follow-up analyses due to the weakness of the statistical evidence, we found nominally significant association of *HGF* gene with the exposure to life-threatening traumatic events in UK Biobank. A recent GEWIS analysis in UK Biobank tested the interaction between traumatic experiences and genetic variation with respect to suicidality, identifying loci involved in brain extracellular matrix biology and synaptic plasticity^50^. The identification of the *HGF* locus appears to be in line with these independent results. Indeed, the protein product of the *HGF* gene is a neurotrophic growth factor that exerts pleiotropic effects on the central nervous system^51^. Further studies will be needed to understand the role of the *HGF* locus in gene-by-environment interactions related to suicidality.

In line with previous studies^4,7,8^, our PRS analysis confirmed the genetic overlap of MD with outcomes related to suicidality spectrum. However, we did not find any interactive effect between MD PRS and polysubstance dependence in the context of SI. This could be due to the fact that the genetic variance of GWAS-identified loci appears to be minimally explained by gene-by-environment interactions^50^. This scenario would support the need of GEWIS to identify alleles responsible to moderate the effect of environmental risk factors.

### Limitations

Although the present study provided novel information regarding the phenotypic and molecular links of polysubstance dependence to suicidality, several limitations should be taken into account while evaluating the novelty of the results presented. Our phenotypic associations highlighted that polysubstance dependence and certain SDs are associated with specific suicidality patterns. Although we accounted for polysubstance comorbidity in our analysis, we did not include covariates related to other psychiatric traits associated with SDs and suicidality (i.e., MD and anxiety). Accordingly, future studies will be needed to understand the effect of psychiatric comorbidities on SD-suicidality associations. We combined genetic and phenotypic data from two large cohorts, but the sample size investigated is not large enough to investigate the polygenic architecture of complex traits like polysubstance dependence and suicidality. To date, there is limited availability of large cohorts informative to investigate SD genetics, especially when considering illegal drugs^39,41^. Although information from Yale-Penn and Army STARRS cohorts were previously combined to replicate genetic associations related to SD and suicidality^8,11^, the limited availability of information regarding polysubstance dependence in Army STARRS participants likely prevented us from replicating the findings observed in the Yale-Penn participants. Additionally, the differences in the demographic characteristics of the two cohorts may also have contributed to reducing the power of our replication analysis. Accordingly, the findings presented will need to be replicated in independent samples that are adequately powered. Finally, accounting for heritable covariates in association tests can lead to spurious associations due to collider bias^52^. Although the StructLMM interaction test is robust to this confounding effect^27^, gene-exposure associations may alter the interpretation of interactions, reflecting epistatic relationships between genetic factors. The loci identified in the present study were not previously identified as associated with substance use, abuse, and dependence. This supports that the interactions identified are not due to epistatic effects.

## Supplementary information

Supplemental Figure 1

Supplemental Table 1

Supplemental Table 2

Supplemental Table 3

Supplemental Table 4

Supplemental Table 5

Supplemental Table 6

Supplemental Table 7

Supplemental Table 8
