# Supplementary material for: Multi-environment gene interactions linked to the interplay between polysubstance dependence and suicidality
Source: Transl Psychiatry. 2021 Jan 11;11:34. doi: 10.1038/s41398-020-01153-1 (PMC7801457; doi:10.1038/s41398-020-01153-1)
Supplement: Supplementary file 1 — Supplemental Figure 1 [file 41398_2020_1153_MOESM1_ESM.docx]

**
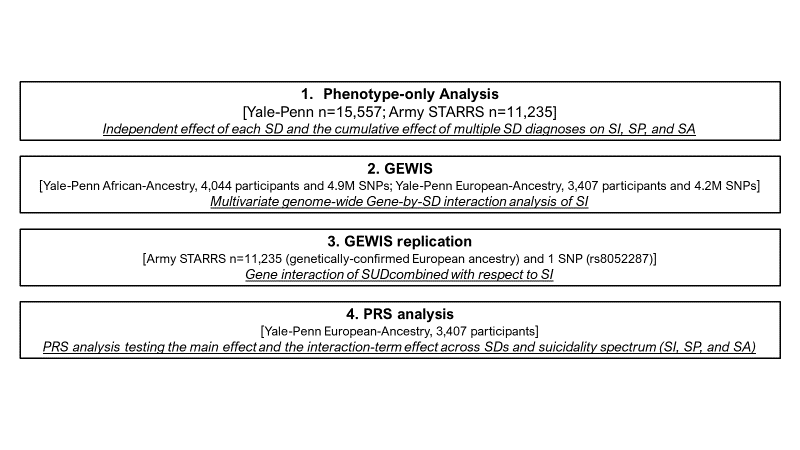
**

**Supplemental Figure 1:** Details regarding each step of the analyses conducted. SD: substance dependence; SI: suicide ideation; SP: suicide planning; SA: suicide attempt; SUD_combined_: a single composite variable combining substance use disorders for alcohol and/or drugs; SNP: single nucleotide polymorphisms.
