# Supplementary material for: Multi-environment gene interactions linked to the interplay between polysubstance dependence and suicidality
Source: Transl Psychiatry. 2021 Jan 11;11:34. doi: 10.1038/s41398-020-01153-1 (PMC7801457; doi:10.1038/s41398-020-01153-1)
Supplement: Supplementary file 2 — Supplemental Table 1 [file 41398_2020_1153_MOESM2_ESM.docx]

**Supplemental Table 1**: Characteristics of the Yale-Penn participants stratified by the inclusion in the GEWIS.

| **Yale-Penn, n=15,557** | **GEWIS** | **Phenotype only** |
| --- | --- | --- |
| Participants, n | 7,451 | 8,106 |
| Age, mean (SD) | 40 (11.2) | 39 (12.3) |
| Sex, Women (%) | 3,259 (44) | 3,928 (48) |
| DSM-IV diagnosis, n (%)  *Alcohol Dependence*  *Cannabis Dependence*  *Cocaine Dependence*  *Nicotine Dependence*  *Opioid Dependence* | 4,116 (55)  2,171 (29)  4,350 (58)  4,290 (58)  2,500 (34) | 3,365 (42)  1,726 (21)  4,312 (53)  3,929 (48)  1,879 (23) |
| Polysubstance Dependence, n (%)  *One DSM-IV SD diagnosis*  *Two DSM-IV SD diagnoses*  *Three DSM-IV SD diagnoses*  *Four DSM-IV SD diagnoses*  *Five DSM-IV SD diagnoses* | 827 (11)  1,422 (19)  1,735 (23)  1,364 (18)  619 (8) | 1,196 (15)  1,520 (19)  1,610 (20)  1,055 (13)  385 (5) |
| Suicidality, n (%)  *Ideation*  *Persistent Ideation*  *Planning*  *Attempt* | 3,343 (45)  807 (11)  1,323 (18)  1,062 (14) | 2,769 (34)  643 (8)  1,168 (14)  903 (11) |
