# Supplementary material for: Multi-environment gene interactions linked to the interplay between polysubstance dependence and suicidality
Source: Transl Psychiatry. 2021 Jan 11;11:34. doi: 10.1038/s41398-020-01153-1 (PMC7801457; doi:10.1038/s41398-020-01153-1)
Supplement: Supplementary file 3 — Supplemental Table 2 [file 41398_2020_1153_MOESM3_ESM.docx]

**Supplemental Table 2**: Characteristics of the Yale-Penn participants stratified by self-reported racial-ethnic groups: Native American/American Indian (NA); Asian (ASN); Pacific Islander (PI); African-American/Black, not of Hispanic origin (AAN); African-American/Black, of Hispanic origin (AAH); Caucasian/White, not of Hispanic origin (CWN); Caucasian/White, of Hispanic origin (CWH).

| **Yale-Penn, total n=15,557** | **NA** | **ASN** | **PI** | **AAN** | **AAH** | **CWN** | **CWH** | **Other** |
| --- | --- | --- | --- | --- | --- | --- | --- | --- |
| Participants, n | 1,327 | 101 | 20 | 6,027 | 350 | 6,060 | 811 | 861 |
| Age, mean (SD) | 32 (8.4) | 30 (9) | 36 (12) | 42 (10.5) | 36 (9.6) | 40 (13.3) | 36 (9.5) | 39 (10.3) |
| Sex, Women (%) | 646 (49) | 44 (44) | 11 (55) | 2,847 (47) | 157 (45) | 2,787 (46 | 320 (39) | 375 (44) |
| DSM-IV diagnosis, n (%)  *Alcohol Dependence*  *Cannabis Dependence*  *Cocaine Dependence*  *Nicotine Dependence*  *Opioid Dependence* | 572 (43)  150 (11)  1,127 (85)  803 (61)  80 (6) | 21 (21)  6 (6)  14 (14)  16 (16)  8 (8) | 9 (45)  4 (20)  7 (35)  7 (35)  2 (10) | 2,982 (49)  1,582 (26)  3,587 (60)  3,039 (50)  1,072 (18) | 180 (51)  111 (32)  209 (60)  207 (59)  128 (37) | 2,861 (47)  1,518 (25) 2,714 (45)  3,166 (52)  2,341 (39) | 413 (51)  258 (32)  485 (60)  486 (60)  409 (50) | 443 (52)  268 (31)  519 (60)  495 (58)  338 (29) |
| Polysubstance Dependence, n (%)  *One DSM-IV SD diagnosis*  *Two DSM-IV SD diagnoses*  *Three DSM-IV SD diagnoses*  *Four DSM-IV SD diagnoses*  *Five DSM-IV SD diagnoses* | 326 (25)  444 (33)  354 (27)  94 (7)  16 (1) | 9 (9)  5 (5)  8 (8)  3 (3)  2 (2) | 3 (15)  6 (30)  2 (10)  2 (10)  0 (0) | 807 (13)  1,252 (21)  1,406 (23)  867 (14)  253 (4) | 39 (11)  66 (19)  80 (23)  75 (21)  25 (7) | 690 (11)  904 (15)  1,117 (18)  1,019 (17)  535 (9) | 66 (8)  113 (14)  181 (22)  179 (22)  100 (12) | 82 (10)  151 (18)  197 (23)  180 (21)  73 (9) |
| Suicidality, n (%)  *Ideation*  *Persistent Ideation*  *Planning*  *Attempt* | 459 (35)  68 (5)  245 (18)  161 (12) | 27 (27)  7 (7)  11 (11)  7 (7) | 6 (30)  2 (10)  5 (25)  4 (20) | 2,093 (35)  410 (7)  836 (14)  674 (11) | 165 (47)  55 (16)  78 (22)  74 (21) | 2,562 (42)  713 (12)  982 (16)  739 (12) | 372 (46)  93 (11)  158 (19)  136 (17) | 427 (50)  102 (12)  176 (21)  170 (20) |
