# Supplementary material for: Multi-environment gene interactions linked to the interplay between polysubstance dependence and suicidality
Source: Transl Psychiatry. 2021 Jan 11;11:34. doi: 10.1038/s41398-020-01153-1 (PMC7801457; doi:10.1038/s41398-020-01153-1)
Supplement: Supplementary file 4 — Supplemental Table 3 [file 41398_2020_1153_MOESM4_ESM.docx]

**Supplemental Table 3**: Association (Odds Ratio, OR, and 95% confidence interval) of self-reported racial-ethnic groups with suicidality outcomes in Yale-Penn participants. The results reported are adjusted for age, sex, and polysubstance dependences.

| **Racial/Ethnic Group** | **Suicidal Ideation** | | | **Persistent**  **Suicidal Ideation** | | | **Suicide Planning** | | | **Suicide Attempt** | | |
| --- | --- | --- | --- | --- | --- | --- | --- | --- | --- | --- | --- | --- |
|  | **OR** | **2.5%** | **97.5%** | **OR** | **2.5%** | **97.5%** | **OR** | **2.5%** | **97.5%** | **OR** | **2.5%** | **97.5%** |
| Native American/American Indian | 0.43 | 0.34 | 0.53 | 0.44 | 0.29 | 0.68 | 1.52 | 1.09 | 2.13 | 0.75 | 0.53 | 1.05 |
| Asian | 0.69 | 0.41 | 1.13 | 0.91 | 0.32 | 2.31 | 1.04 | 0.42 | 2.41 | 0.48 | 0.15 | 1.25 |
| Pacific Islander | 0.55 | 0.16 | 1.61 | 2.29 | 0.27 | 19.64 | 6.67 | 0.95 | 132.8 | 2.39 | 0.38 | 18.88 |
| African-American/Black, not of Hispanic origin | 0.50 | 0.41 | 0.59 | 0.74 | 0.55 | 0.99 | 0.99 | 0.77 | 1.27 | 0.72 | 0.56 | 0.93 |
| African-American/Black, of Hispanic origin | 0.82 | 0.61 | 1.12 | 1.89 | 1.19 | 3.01 | 1.47 | 0.97 | 2.24 | 1.27 | 0.82 | 1.94 |
| Caucasian/White, not of Hispanic origin | 0.74 | 0.62 | 0.88 | 1.17 | 0.88 | 1.56 | 1.01 | 0.79 | 1.29 | 0.69 | 0.54 | 0.89 |
| Caucasian/White, of Hispanic origin | 0.78 | 0.61 | 0.99 | 1.24 | 0.85 | 1.81 | 1.26 | 0.91 | 1.76 | 1.01 | 0.72 | 1.41 |
