# Supplementary material for: Multi-environment gene interactions linked to the interplay between polysubstance dependence and suicidality
Source: Transl Psychiatry. 2021 Jan 11;11:34. doi: 10.1038/s41398-020-01153-1 (PMC7801457; doi:10.1038/s41398-020-01153-1)
Supplement: Supplementary file 6 — Supplemental Table 5 [file 41398_2020_1153_MOESM6_ESM.docx]

**Supplemental Table 5**: Association of rs8052287 surviving Bonferroni multiple testing correction in the phenome-wide scan conducted in the GWAS atlas.

| **atlas ID** | **PMID** | **Year** | **Domain** | **Trait** | **P-value** | **N** |
| --- | --- | --- | --- | --- | --- | --- |
| 4043 | 30124842 | 2018 | Skeletal | Height | 8.30E-22 | 693529 |
| 3187 | 31427789 | 2019 | Skeletal | Standing height | 4.34E-18 | 385748 |
| 4352 | 30573740 | 2018 | Dermatological | Male pattern baldness | 9.10E-13 | 205327 |
| 3503 | 31427789 | 2019 | Dermatological | Hair/balding pattern: Pattern 4 | 1.11E-10 | 176380 |
| 3271 | 31427789 | 2019 | Skeletal | Comparative height size at age 10 | 1.14E-10 | 380167 |
| 4280 | 30804560 | 2019 | Respiratory | FEV1/FVC ratio | 5.03E-10 | 400102 |
| 4080 | 30239722 | 2018 | Metabolic | Waist-hip ratio (adjusted for BMI) | 1.51E-09 | 694649 |
| 4226 | 31015401 | 2019 | Environmental | Thyroid preparations | 1.20E-08 | 305582 |
| 4175 | 30048462 | 2018 | Skeletal | Heel bone mineral density | 6.40E-08 | 394929 |
| 4328 | 30598549 | 2018 | Skeletal | Estimated bone mineral density from heel ultrasounds | 9.90E-08 | 426824 |
| 3887 | 27863252 | 2016 | Immunological | Mean corpuscular hemoglobin | 4.74E-07 | 172332 |
| 3602 | 31427789 | 2019 | Endocrine | hypothyroidism/myxoedema | 5.97E-07 | 289307 |
| 3448 | 31427789 | 2019 | Metabolic | Impedance measures - Impedance of leg | 1.25E-06 | 379813 |
| 3851 | 27863252 | 2016 | Immunological | Mean corpuscular hemoglobin | 1.28E-06 | 132224 |
| 173 | 25673412 | 2015 | Metabolic | Waist circumference (adjusted for BMI) | 1.60E-06 | 231355 |
| 3848 | 27863252 | 2016 | Immunological | Lymphocyte count | 1.92E-06 | 132452 |
| 3852 | 27863252 | 2016 | Immunological | Mean corpuscular volume | 1.93E-06 | 132353 |
| 3500 | 31427789 | 2019 | Dermatological | Hair/balding pattern: Pattern 1 | 3.27E-06 | 176380 |
| 3884 | 27863252 | 2016 | Immunological | Lymphocyte count | 3.30E-06 | 171643 |
| 3891 | 27863252 | 2016 | Immunological | Mean platelet volume | 7.05E-06 | 164454 |
| 3310 | 31427789 | 2019 | Activities | Age started wearing glasses or contact lenses | 8.61E-06 | 332074 |
