# Supplementary material for: Multi-environment gene interactions linked to the interplay between polysubstance dependence and suicidality
Source: Transl Psychiatry. 2021 Jan 11;11:34. doi: 10.1038/s41398-020-01153-1 (PMC7801457; doi:10.1038/s41398-020-01153-1)
Supplement: Supplementary file 8 — Supplemental Table 7 [file 41398_2020_1153_MOESM8_ESM.docx]

**Supplemental Table 7**: Association of major depression polygenic risk score with suicidal behaviors in the Yale-Penn cohort. Across the suicide behaviors tested, PT=0.5 (in bold) showed the best results. Results surviving false discovery rate multiple testing correction (FDR q<0.05) are underlined. MD: Major Depression; PRS: Polygenic Risk Score; PT: P-value Threshold; SNP: single nucleotide polymorphism; OR: Odds Ration; CI2.5%: Lower Bound of 95% Confidence Interval; CI97.5%: Upper Bound of 95% Confidence Interval.

| **MD PRS** | **SNP, n** | **Suicide Ideation** | | | **Persistent Suicide Ideation** | | | **Suicide Planning** | | | **Suicide Attempt** | | |
| --- | --- | --- | --- | --- | --- | --- | --- | --- | --- | --- | --- | --- | --- |
| *PT* |  | *OR* | *CI2.5%* | *CI97.5%* | *OR* | *CI2.5%* | *CI97.5%* | *OR* | *CI2.5%* | *CI97.5%* | *OR* | *CI2.5%* | *CI97.5%* |
| 5.E-08 | 12 | 1.00 | 0.91 | 1.09 | 1.02 | 0.88 | 1.17 | 0.97 | 0.86 | 1.10 | 1.04 | 0.91 | 1.19 |
| 1.E-07 | 15 | 1.05 | 0.96 | 1.14 | 1.09 | 0.95 | 1.25 | 1.06 | 0.94 | 1.20 | 1.05 | 0.92 | 1.20 |
| 1.E-06 | 35 | 1.06 | 0.97 | 1.16 | 1.14 | 0.99 | 1.30 | 1.06 | 0.93 | 1.20 | 0.96 | 0.84 | 1.09 |
| 1.E-05 | 97 | 1.08 | 0.99 | 1.18 | 1.11 | 0.96 | 1.27 | 1.09 | 0.96 | 1.23 | 1.01 | 0.88 | 1.16 |
| 1.E-04 | 348 | 1.07 | 0.98 | 1.17 | 1.06 | 0.93 | 1.22 | 1.00 | 0.88 | 1.13 | 0.95 | 0.83 | 1.09 |
| 0.001 | 1,548 | 1.06 | 0.97 | 1.15 | 1.17 | 1.02 | 1.34 | 1.01 | 0.89 | 1.14 | 1.02 | 0.90 | 1.17 |
| 0.05 | 26,228 | 1.08 | 0.99 | 1.19 | 1.17 | 1.02 | 1.36 | 1.16 | 1.02 | 1.32 | 1.16 | 1.01 | 1.33 |
| 0.1 | 42,861 | 1.09 | 1.00 | 1.20 | 1.19 | 1.03 | 1.37 | 1.22 | 1.08 | 1.39 | 1.19 | 1.04 | 1.36 |
| 0.3 | 89,867 | 1.10 | 1.00 | 1.21 | 1.24 | 1.07 | 1.43 | 1.26 | 1.11 | 1.44 | 1.25 | 1.09 | 1.44 |
| ***0.5*** | ***120,987*** | ***1.09*** | ***0.99*** | ***1.21*** | ***1.26*** | ***1.09*** | ***1.46*** | ***1.28*** | ***1.13*** | ***1.47*** | ***1.26*** | ***1.09*** | ***1.45*** |
| 1 | 157,478 | 1.09 | 0.99 | 1.20 | 1.25 | 1.08 | 1.45 | 1.28 | 1.12 | 1.46 | 1.25 | 1.08 | 1.44 |
