# Supplementary material for: Multi-environment gene interactions linked to the interplay between polysubstance dependence and suicidality
Source: Transl Psychiatry. 2021 Jan 11;11:34. doi: 10.1038/s41398-020-01153-1 (PMC7801457; doi:10.1038/s41398-020-01153-1)
Supplement: Supplementary file 9 — Supplemental Table 8 [file 41398_2020_1153_MOESM9_ESM.docx]

**Supplemental Table 8:** Interaction between major depression polygenic risk score and substance dependences with respect to suicidal behaviors in Yale-Penn cohort. The analysis was restricted to the combinations of suicidal behaviors and substance dependences significantly associated with each other (Figure 1). MD: Major Depression; PRS: Polygenic Risk Score; OR: Odds Ration; CI2.5%: Lower Bound of 95% Confidence Interval; CI97.5%: Upper Bound of 95% Confidence Interval; na: not applicable.

| **Variable** | **Suicide Ideation** | | | **Persistent Suicide Ideation** | | | **Suicide Attempt** | | |
| --- | --- | --- | --- | --- | --- | --- | --- | --- | --- |
|  | *OR* | *CI2.5%* | *CI97.5%* | *OR* | *CI2.5%* | *CI97.5%* | *OR* | *CI2.5%* | *CI97.5%* |
| Alcohol Dependence (AD) | 2.57 | 2.03 | 3.25 | 1.69 | 1.10 | 2.62 | 1.84 | 1.22 | 2.83 |
| Cocaine Dependence | 1.15 | 0.90 | 1.46 | 0.95 | 0.66 | 1.37 | 1.38 | 0.97 | 1.98 |
| Cannabis Dependence (CaD) | 1.74 | 1.40 | 2.17 | 1.41 | 1.02 | 1.96 | 0.82 | 0.60 | 1.12 |
| Opioid Dependence | 1.01 | 0.80 | 1.27 | 0.96 | 0.69 | 1.35 | 1.09 | 0.79 | 1.50 |
| Nicotine Dependence (ND) | 1.37 | 1.10 | 1.71 | 0.86 | 0.60 | 1.23 | 1.14 | 0.81 | 1.62 |
| MD PRS | 1.09 | 0.92 | 1.29 | 1.36 | 0.99 | 1.88 | 1.33 | 0.96 | 1.84 |
| MD PRS × AD | 1.04 | 0.84 | 1.28 | 1.03 | 0.71 | 1.50 | 0.94 | 0.66 | 1.33 |
| MD PRS × CaD | 0.86 | 0.70 | 1.05 | 0.81 | 0.60 | 1.10 | na | na | na |
| MD PRS × ND | 1.06 | 0.87 | 1.30 | na | na | na | na | na | na |
